# Cancer in Patients Referred Abroad For Health Care and Related Foreign Currency Expenses

**DOI:** 10.24248/eahrj.v5i2.668

**Published:** 2021-11-15

**Authors:** Ruben Niyonsaba, Astère Manirakiza, Laurent Irakoze

**Affiliations:** a Hope Africa university, Department of nursing sciences; b Teaching University Hospital of Kamenge

## Abstract

**Background:**

There is limited access to health services in Burundi, as most of the services such as cancer care are unavailable. Burundian citizen who can afford the costs involved in seeking treatment elsewhere are referred abroad. The purpose of this study was to assess the proportion of patients suffering from cancer among patients referred abroad for healthcare and to evaluate the costs incurred by those patients in relation to what the country would save by establishing cancer healthcare facilities.

**Methodology:**

The study was performed retrospectively from January 2016 to December 2018. With approval of Ministry of Public Health and AIDS control, the data was collected from medical reports at the general management of health facilities and AIDS control office. All patients with medical reports containing the reason for referral were included in the study. Medical reports assessing occupational disability were excluded. Data analysis was performed using Statistical Package for the Social Sciences (SPSS).

**Results:**

Male, female and unclear was 45.3%, 39.9% and 14.8% respectively. Average age was 31,82. The main reason for referral was MRI (21.7%). Cancer patients represented 18% of all patients referred abroad for healthcare and the most common type of cancer found was breast (26.5%), genitourinary (15.7%) and digestive (14,2%). If all patients from 2016-2018 were referred to Kenya, Uganda, Rwanda, India or Europe for 30 years, the country would spend in foreign currency US$3,858,229; US$638,342.80; US$21,288,592; US$10,410,192.90; US$54,718,329.70 respectively. Also, if all patients estimated by Globocan in 2018 were to be referred to these countries, the cost of foreign currencies would be US$52,455,122.60; US$38,264,740.88; US$129,272,590.40; US$81,330,325.94; US$276,601,008.02 respectively.

**Conclusion:**

There is a good number of cancer patients among patients referred abroad for health care. The estimated costs incurred by patients referred abroad for cancer care are far greater than funds needed to setup modern cancer care centres in Burundi.

## BACKGROUND

Worldwide, 19.3 million new cancer cases and 10 million deaths from cancer were recorded in the year 2020. In Africa, the incidence was estimated to be 5.7% of worldwide incidence while death from cancer was rated at 7.2% of the total worldwide deaths. Of these, 5.7% deaths due to cancer were estimated to occur in Africa, with breast cancer overtaking lung cancer as the most frequently diagnosed cancer type with approximately 2.3 million new cases (11.7%), followed by lung cancer (11.4%), colorectal (10.0%), prostate (7.3%) and stomach cancers (5.6%).^1^

In Burundi, the incidence of cancer was estimated to be 6,743; 8,682 and 7,929 new patients respectively for the year 2012, 2018 and 2020.^2–4^ However, this data is estimated using information from neighbouring countries since Burundi does not have a cancer incidence registry.

Cancer care in Burundi remains a challenge. Indeed, accessing pathological diagnosis and chemotherapy is problematic since there is only one pathologist for every 12 million people, 3 Computed Tomography (CT) scan, no Magnetic Resonance Imaging (MRI) and no chemotherapy in public facilities.

Due to difficulties in accessing advanced healthcare such as MRI or pathologic tests in Burundi, the cancer prevalence could have been underestimated.

According to the World Health Organization, the right to health for all people means that everyone should have access to health facilities they need, when and where they need them without suffering financial hardship.^5^ Unfortunately, most cancer patients in Burundi have to be referred abroad to be able to access advanced healthcare services. However, seeking treatment from abroad comes with huge expenses which only a few can afford. The cancer diagnosis and treatment dilemma requires urgent action so as to help people suffering from cancer.^6^

### Study Questions

In the framework of advocating for the population so that health authorities in Burundi realise the ampleness of the problem, this study was conducted with the following study questions: (1) What is the proportion of cancer among patients referred abroad for healthcare, (2) Is there any benefit that the country could register if advanced cancer healthcare services were provided within Burundi?

## METHODS

### Process of Getting Authorisation to be Treated Abroad

For patients whose doctors recommends seeking healthcare abroad or to assess occupational disability, the doctors writes to the Ministry of Public Health and the Fight against AIDS requesting the ministry to appoint a medical commission to assess the need for such a patient to be treated abroad or to assess occupational disability of the patient. The appointed commission studies the patient's situation and makes a report recommending whether the request is founded or not. The report is forwarded to the general manager of public health facilities who, basing on the commission's recommendations authorises or rejects the request for seeking treatment abroad.

### Consultation of Medical Reports

The study was conducted at the Ministry of Public Health and AIDS control. Permission to conduct the study was sought for from the Ministry Of Public Health and AIDS by Hope Africa University of Burundi.

Data was collected from the office of the General Manager of public health facilities, a department in charge of nomination of the Medical Committee, a Commitee that assesses the need for patients to seek health services abroad.

### Inclusion and Exclusion Criterions

The study assessed all submitted medical reports of January 2016 to December 2018. To be included in the study, the report had to be mentioning Cancer as the reason for referral and diagnosis. Medical reports assessing other issues such as occupational disability were excluded.

### Data Collection and Analysis

Data was extracted from the medical reports. After extraction, the information was recorded on a structured questionnaire developed for this purpose. This questionnaire contained variables such as; age, gender, hospital producing medical report, diagnosis, reason and year of referral, medical specialty, cancer type and MRI exam. The recorded data was registered, analysed and transferred into SPSS 25.0 (IBM Corp, Armonk, NY). Univariate descriptive analysis was performed.

### Online Searching

Extensive search was done on Google scholar, PubMed, and other website to compare different price of oncology facilities, life and travel cost in the countries where most of Burundian travel to seeking cancer healthcare. Results were combined to determine the average of the personal expenses incurred. The average was applied to all the number of patients that were referred abroad to seek treatment for cancer. Results were extrapolated, patients of 3 to 10 years on one hand and all cancer patients estimated by Globocan in 2018 to be referred abroad for treatment on the other hand. The total sums were compared with the amount required to set up a Cancer care centre.

### Ethical Approval

Through the Hope Africa University, a letter was sent (Réf:UEA/PPU/216/12/2018) to the Ministry of Public Health and of AIDS requesting for authorisation to consult the medical reports produced by Medical Commissions in charge of assessing the necessity of referral of patients to seek treatment abroad. The request was approved through a letter (N° 633/738/DGSSLS/2018).

### RESULTS

From January 2016 to December 2018, 1,172 patients requested medical reports for approval of referral to seek for treatment abroad. During the screening process, 104 of these were excluded because they did not fulfil the criteria of inclusion. 1,068 medical records were analysed (Figure 1).

### Socio-Demographic Characteristics and Reason for Referral

Patients were grouped into 3 groups; male, female and not clear (for applications from couples without any precision about who is seeking the health care). Male, female and not clear represented 484(45.3%), 426(39.9%) and 158(14.8%) respectively (Table 1).

**TABLE 1:** Socio-Demographic Characteristics

Variables	Frequency (N=370)	Percent (%)
**Gender**		
Male	484	45.3
Female	426	39.9
Unclear	158	14.8
**Age grouping (1068)**		
Under 16 years	208	19.5
16 – 49	298	27.9
50 – 76	150	14.0
Not mentioned	412	38.6
**Hospital producing medical report (1068)**		
Teaching hospital of Kamenge	466	43.6
Military Hospital of Kamenge	201	18.8
Prince Louis Rwagasore Clinic	78	7.3
Kira Hospital	69	6.5
Prince Regent Rwagasore hospital	87	8
Others hospitals	129	12.2
Not mentioned	38	3.6

The average age of referred patients was 32.8, ranging between 16 to 49 years, 298(27.9%). However, in many medical records, age was not mentioned 412(38.6%). Hospitals which referred most of the patients to the committee were University Teaching Hospital of Kamenge 466(43.6%) and Military hospital of Kamenge 201(18.8%) (Table 1).

Of the referred patients, 197(18.4%) were referred because of cancer. Of these; 52(26.5%), 31(15.7%), 28(14.2%) and 28(14.2%) represented breast, genito-urinary, Oto-rhino-laryngological and hepato-gastroentological cancer respectively (Table 2).

**TABLE 2:** Place of Tumour and the Main Reasons of Referral

Variables	Frequency (N=1068)	(%)
**Tumours**		
Yes	197	18.4
No	871	81.6
**Main tumour site**	**Frequency** **(N=197)**	(%)
Breast	52	26.5	
Genito-Urinary	31	15.7
Brain and neurological	21	10.7
Oto rhino laryngological	28	14.2
Homeopathy	6	3.0	
Hepato-Gastroentology	28	14.2
Lungs Cancer	4	2.0
Others	27	13.7
**Magnetic resonance imaging**	**Frequency (N=1068)**	(%)
Yes	232	21,7
No	836	79,3
**Group of main reasons of transfer**	**Frequency** **(N=1068)**	(%)
Tests	404	37.8
Medical care	294	27.6
MAP (Medical assisted Procreation)	156	14.6
Cardiac surgery	125	11.7
Others	89	8.3

Magnetic Resonance Imaging (MRI) was the main reason for seeking approval to travel abroad 232(21,7%). In general, most of patients were referred abroad to carry out tests 404(37.8%) (Table 2).

Considering the affected organs including cancer and other diseases, neurological 238(22.3%), Genitourinary 233(21.8%) and cardiovascular system 172(16.1%) were the mostly affected among referred patients (Table 3). Many patients were referred in the year 2018, 431(40,4%) (Table 4) and many cancer patients were referred in the year 2018 (40,6%) (Table 5). Although in most cases the patient's age was not mentioned, majority of referred patients were over 50 years old (Table 5).

**TABLE 3:** Frequency of Patients According to Affected Sites among Referred Patients and Year Of Referral

Affected sites	Frequency (1068)	%
Neurological diseases	238	22.3
Cardiovascular diseases	172	16.1
Endocrinology	23	2.2
Rheumatology	20	1.9
Hepato-Gastroenterology	54	5.1
Surgery	88	8.2
Genito-urinary diseases	233	21.8
Oto rhino laryngological	71	6.6
Dermatology	15	1.4
Ophthalmology	49	4.6
Haematology	19	1.8
Lungs diseases	10	0.9
Breast diseases	52	4.9
Nephrology	15	1.4
Others	9	0.8
**Year of transfer**		
2016	267	25,0
2017	370	34,6
2018	431	40,4

**TABLE 4:** Frequency of Cancer Patients According to Transfer Year, Age and Gender

Year of transfer	Cancer (197)	
	Number	%
2016	43	21,8
2017	74	37,6
2018	80	40,6
**Age grouping**		
< 16	22	10.6
≥ 16 – 49	83	27.9
≥50	45	30.0
Not mentioned	47	31,5
**Gender**		
Male	78	39,6
Female	119	60,4

**TABLE 5:** Estimated Cost of Cancer Treatment, Transport and Living Expenses for 5 Frequently Visited Countries

Country	Cost of Cancer Care(US$)		Travel fees & references(US$)	Living Expenses for 4 months & refernces	Individual Cost(US$)	Gross domestic domestic Product per Capita & references
**Kenya** ^7^	476,5	Diagnostic fees	400^8^	2060^9^	7240,30	3483^10^
	1643,4	Surgery				
	1,364.3	Chemotherapy				
	1,296.1	Radiotherapy				
	1,716.4	Chemo-radiotherapy				
**Rwanda** ^11,12^	1524,36	Without radiotherapy	228,48^8^	346,94^13^	3512,84	2165^10^
	2093	Radiotherapy				
**Uganda** ^14^	4335	Surgery	320^8^	2076,20^15^	16088,20	2183^10^
	9360	Chemo-radiotherapy				
**India** ^16^	502	Cost of investigation	825,37^17^	1480,80^18^	10556,17	8443^19^
	1386	Chemotherapy				
	6382	Radiotherapy				
**Europe**	28624,75	Surgery	636,86^8^	3796^20^	33057,61	34768.65^21^
**Burundi**						732^10^

### The Average Cost of Expenses Related to Cancer Care, Travel and Subsistence Expenses in Countries where Burundians are Often Referred to

Extensive online searching was performed. General expenses related to cancer care, living expenses and travel in countries where Burundian patients are often referred to were identified. The countries included; Kenya, Uganda, Rwanda, India and Belgium (Table 5). Individual average expenses were summarised for each of the country; US$7,240.30; US$3,512.84; US$16,088.20; US$10,556.17 and US$33,057.61 in Kenya, Rwanda, Uganda, India and in Europe respectively. (Table 5a).

In this study, we analysed the expenses for all patients referred abroad for cancer healthcare services and facilities during 3 years, 30 years and if all new cases estimated in 2018 where to be referred abroad (Table 6). In this analysis, it is clear that Burundi expends large amounts of foreign currencies to countries where patients are referred. Indeed, if all patients were referred to Kenya, Uganda, Rwanda, India or Europe for a period of 30 years, the country would be spending in foreign currency US$3,858,229.00; US$638,342.80; US$21,288,592.00, US$10,410,192.90, US$54,718,329.70 respectively.

**TABLE 6:** Expense for Patients Referred Abroad and Money to be Saved if Cancer Care Facility were Established in Burundi

Country	Average of individual expenses(US$)	Expenses incurred by Cancer patient referred abroad	Extrapolation of expenses on 30 yrs (US$) (2016-2018) (US$)	Expenses to set up a centre of oncology & reference	Loss of money within 30 yrs	If all patients estimated by Globocan in 2018 where referred abroad (8682)	Difference if all patients estimated by Globocan in 2018 should be referred abroad
Kenya	7,240.30	1,426,339.10	14,263,391.00		3,858,229.00	62,860,284.60	52,455,122.60
Uganda	5,605.84	1,104,350.48	11,043,504.80		638,342.80	48,669,902.88	38,264,740.88
Rwanda	16,088.20	3,169,375.40	31,693,754.00	10,405,162^22^	21,288,592.00	139,677,752.40	129,272,590.40
India	10,566.17	2,081,535.49	20,815,354.90		10,410,192.90	91,735,487.94	81,330,325.94
Europe	33,057.61	6,512,349.17	65,123,491.70		54,718,329.70	287,006,170.02	276,601,008.02

If all patients estimated by Globocan in 2018 were referred to Kenya, Uganda, Rwanda, India or Europe, the loss of foreign currencies if Burundi was to establish a cancer centre whose value is estimated at 11 million American dollars is; US$52,455,122.60; US$38,264,740.88; US$129,272,590.40; US$81,330,325.94 and US$276,601,008.02 respectively.

## DISCUSSION

This descriptive study assessed the frequency of cancer among patients referred abroad to seek access to better health services after seeking for government authorisation. In addition, it shows the main reasons for the referrals and the related foreign currency expenses.

The results of this study can be used to estimate the rate of cancer among patients referred abroad for healthcare, to estimate the foreign currency expenses that the country could earn if cancer health care services were introduced in Burundi and can form a key advocacy for improving scope of health services offered in Burundi.

Of all patients referred abroad for cancer treatment, about 18% of these patients actually travelled and the rate could be lower given the realities on ground. In fact, many of the patients are unable to afford the costs involved to access health services abroad. Some of the patients will never know that they suffer from cancer due to lack of cancer diagnosis services in Burundi.

There is scanty data about patients who were referred abroad for cancer treatment but did not go. If such data was available, it would help estimate the demand for cancer treatment services among patients that are referred abroad.

Considering data estimates by Globocan in 2018, a good number of cancer patients in Burundi did not access treatment. Indeed, 8,682 new cancer patients were expected to occur in 2018 in Burundi^23^ but only 80 patients were referred abroad for diagnosis and treatment. Since there was no cancer care centre in Burundi at that time, it implies that approximately 8,602 patients did not received cancer care.

In other countries within the East African Community, there is also scanty data about patients referred abroad for cancer healthcare. The reason could be that the quality of their healthcare is better than that of Burundi.

Among the patients refereed abroad, a big percentage of them were suffering from cancer. This shows that universal healthcare coverage is a challenge in Burundi.

In fact, most of the patients referred abroad were being sent to perform medical tests, medical assisted procreation and cardiac surgery etc. This shows the need for efforts to equip health facilities with necessary machinery and also to train health personnel to meet the health needs of the population.

The government of Burundi does not have any strategy in place to follow up on the outcome of patients that are referred abroad to receive healthcare; Did the patients receive the sought for care or not? Such data is missing. Such data would help to evaluate the impact of health services administered abroad and facilitate policymaking. For instance, in Senegal, among 169 children referred abroad for health services especially cardiac surgery, only 48.8% had good results and the quality of life was better in the group of congenital heart disease.^5^

This study showed that cancers were associated with gender and age. In fact, breast cancer is the most type of cancer found in the world and in Africa, cancer is supposed to be a disease of old age.^4,5^ The tendency of world cancer statistics estimated that female victims to cancer were to be around 47.5% of all new cancer incidents in 2018.^6^

This study reports that the cost (treatment, travel and living expenses) of seeking for cancer healthcare in the 5 frequented countries remains high irrespective of the country in which treatment is sought. Therefore, we note that during the 3 years of this study, the country lost more foreign currency by engaging in referral of patients abroad (197 patients) for diagnosis and/or treatment of cancer. Extrapolated on 30 years or considering that all patients estimated by Globocan 2018 were referred abroad for treatment, the total sum of foreign exchange is far greater than the amount needed to establish a Cancer Care Centre in Burundi.

Therefore, a good plan should be put in place aimed at saving foreign currency spent by patients seeking diagnosis and treatment of cancer abroad. This will also make it easier for cancer patients to access treatment locally. Other countries such as Benin and Côte d’Ivoire have discovered this inequality and have already made the decision to equip their health centres instead of referrals abroad.^7^

This study has some limitations, limitations related to retrospective studies. Furthermore, there was no standard format of the medical reports used by the Commission. This limited accessibility to some data. Also, this study did not consider the transfer of samples, this brings about bias in the overall tendency of prevalence of cancer.

## CONCLUSION

A good number of patients referred abroad for healthcare between January 2016 and December 2018 were suffering from cancer. Most patients referred abroad for para clinical exams were seeking MRI services. By referring patients abroad for healthcare, the country lose a lot of foreign exchange which would be saved if the government was to equip its medical facilities. The estimated revenue spent by patients referred abroad for treatment is far greater than the funds required to establish a modern Cancer Care Centre. The health authorities could invest in establishing modern Cancer Care Centres in Burundi rather than supporting patient referrals abroad. This is also valid for other missing types of healthcare in Burundi such cardiac surgery. Further studies are required to clarify the situation regarding cancer status among patients requesting health facilities abroad and their follow up.
